# Human Health Risk Assessment of the Photocatalytic Oxidation of BTEX over TiO_2_/Volcanic Glass

**DOI:** 10.3390/molecules28248119

**Published:** 2023-12-15

**Authors:** Madi Smaiyl, Yerzhigit Tulebekov, Nurbek Nurpeisov, Bagdat Satybaldiyev, Daniel D. Snow, Bolat Uralbekov

**Affiliations:** 1 Center of Physical-Chemical Methods of Research and Analysis, Al-Farabi Kazakh National University, Almaty 050012, Kazakhstan; smaiylmadi.9999@gmail.com (M.S.); yerjigit@gmail.com (Y.T.); nurpi@mail.ru (N.N.); bagdat.satybaldiev@gmail.com (B.S.); 2 LLP «EcoRadSM», Almaty 050000, Kazakhstan; 3 Water Sciences Laboratory, Nebraska Water Center, Part of the Daugherty Water for Food Global Institute, University of Nebraska, Lincoln, NE 68583, USA; dsnow1@unl.edu

**Keywords:** photocatalytic oxidation, BTEX, TiO_2_ photocatalyst, hazard index

## Abstract

This study demonstrates rapid photocatalytic oxidation of a benzene, toluene, ethylbenzene, and xylene (BTEX) mixture over TiO_2_/volcanic glass. The assessment of the photocatalytic oxidation of BTEX was conducted under conditions simulating those found in indoor environments affected by aromatic hydrocarbon release. We show, under UV-A intensities of 15 mW/cm^2^ and an air flow rate of 55 m^3^/h, that low ppmv levels of BTEX concentrations can be reduced to below detectable levels. Solid-phase microextraction technique was employed to monitor the levels of BTEX in the test chamber throughout the photocatalytic oxidation, lasting approximately 21 h. Destruction of BTEX from the gas phase was observed in the following sequence: *o*-xylene, ethylbenzene, toluene, and benzene. This study identified sequential degradation of BTEX, in combination with the stringent regulatory level set for benzene, resulted in the air quality hazard indexes (Total Hazard Index and Hazard Quotient) remaining relatively high during the process of photocatalytic oxidation. In the practical application of photocatalytic purification, it is crucial to account for the slower oxidation kinetics of benzene. This is of particular importance due to not only its extremely low exposure limits, but also due to the classification of benzene as a Group 1 carcinogenic compound by the International Agency for Research on Cancer (IARC). Our study underscores the importance of taking regulatory considerations into account when using photocatalytic purification technology.

## 1. Introduction

Air pollution, characterized by a diverse range of compounds, continues to pose a significant threat to human health—particularly in urban and industrial settings [1,2]. Among the major pollutants are sulfur and nitrogen oxides, volatile organic compounds (VOCs), and particulate matter, all of which contribute significantly to air pollution [3]. Aromatic hydrocarbons such as benzene, toluene, ethylbenzene, and xylene (collectively known as BTEX) are among the most prevalent in the atmosphere of urban and industrial areas [4].

Photocatalytic oxidation (PCO) has gained prominence as a technology for effectively removing gaseous pollutants. PCO techniques involve the excitation of an electron from the valence band to the conduction band, which occurs when the energy of the absorbed photon surpasses the band gap of the semiconductor material [5,6]. However, there are noteworthy drawbacks associated with this method, with one of the primary limitations being the incomplete oxidation of organic compounds on the catalyst’s surface. Incomplete oxidation may potentially lead to the production of more harmful by-products in the gas phase than the original pollutants themselves [7,8].

In the realm of photocatalysis, it is important to acknowledge that most studies are centered around the treatment of a single compound. A limited body of research has focused on the photocatalytic oxidation of mixtures likely to be associated with actual human health risks. Mahmood et al. demonstrated a reduced degradation efficiency of benzene (10%) when it was present in a mixture with benzene and p-xylene, compared to its isolated mode degradation (67%). As shown by [9], the photocatalytic treatment of VOC mixtures cannot be directly extrapolated from single VOC behavior even at the ppb level. Of the studies available, only a few have actively monitored potential hazards using hazard indexes such as hazard quotients (HQ) during the photocatalytic oxidation of aromatic compounds [10,11,12]. In these studies, relatively high exposure limit values derived for workplace settings are used to calculate HQ. Furthermore, these assessments are conducted under static conditions and with elevated initial VOC concentrations. Therefore, the feasibility of utilizing PCO air purification technology for indoor applications remains an unresolved matter [13].

In this study, we measured air quality changes during PCO under simulated indoor conditions that started at BTEX concentrations near 1 ppmv. A detailed assessment of the BTEX decomposition in the test chamber using solid phase microextraction provided clear evidence of its oxidation rates, and allows us to show a temporal change in risk from inhalation. BTEX compounds have garnered recent attention due to their adverse effects on both the environment, including the formation of photochemical smog, and human health, with potential links to various diseases including cancer [4,8,14].

## 2. Results and Discussion 

### 2.1. Catalyst and Catalyst Support 

The indexed diffraction peaks at 25.3, 37.8, 48.0, 53.9, 55.1, 62.7, and 71.1° correspond to the (011), (004), (020), (015), (121), (024), and (125) planes, matching well with the tetragonal structure of anatase (TiO_2_), with a I41/amd space group (a = 3.7855(7) Å, c = 9.5122(6) Å)) and peaks at 27.4, 36.1, 41.2, and 54.3 corresponding to the (110), (101), (111), and (211) planes of the rutile (TiO_2_) tetragonal structure (P 42/mnm space group, a = 4.5952(9) Å, c = 2.9572(6) Å), as demonstrated by the Le Bail refinement method shown in Figure S1. Photocurrent measurements for the TiO_2_ powder used, as reported in a separate source [15], demonstrated a rapid increase in photocurrent upon the activation of light and a subsequent decrease to zero when the light was switched off, indicating that the material exhibits a reproducible response to light.

The ideal catalyst support material should possess several key properties, including a high surface area, excellent transparency, a porous structure, strong adsorptive affinity for VOCs, and stability when exposed to UV irradiation [16]. Moreover, thermal stability is a crucial consideration, given that the photocatalyst regeneration process involves calcination. In this study, the chosen catalyst support was volcanic glass, a mixture of alumosilicate minerals, as confirmed by the Le Bail refinement method (as depicted in Figure S2). Thermal analysis TG/DSC revealed that the catalyst support remained stable from room temperature to 1000 °C, showing no discernible phase transitions in this temperature range (Figure S3). A minor endothermic peak was observed in the DSC curve at temperatures below 100 °C, extending to ca. 150 °C. This peak was associated with small water evaporations and the dehydration process. No significant weight loss was detected within the specified temperature range. Furthermore, an IR spectrum was acquired for a sample of the volcanic glass substrate across wavenumber values ranging from 450 to 4000 cm^−1^ (refer to Figure S4). Volcanic glass, being a component of volcanic rocks, primarily consists of silicates and aluminosilicates. Peaks in the range of 1000–1200 cm^−1^ correspond to stretching vibrations of Si–O and Al–O bonds and bands within the regions of 450–600 cm^−1^ and 700–900 cm^−1^ are associated with stretching vibrations, while bands around 1600 cm^−1^ and 2300 cm^−1^ are attributed to the asymmetric stretching of Si-O bonds in the silicate group [17,18,19].

### 2.2. Recirculation Test in the Chamber

**Figure 1:**
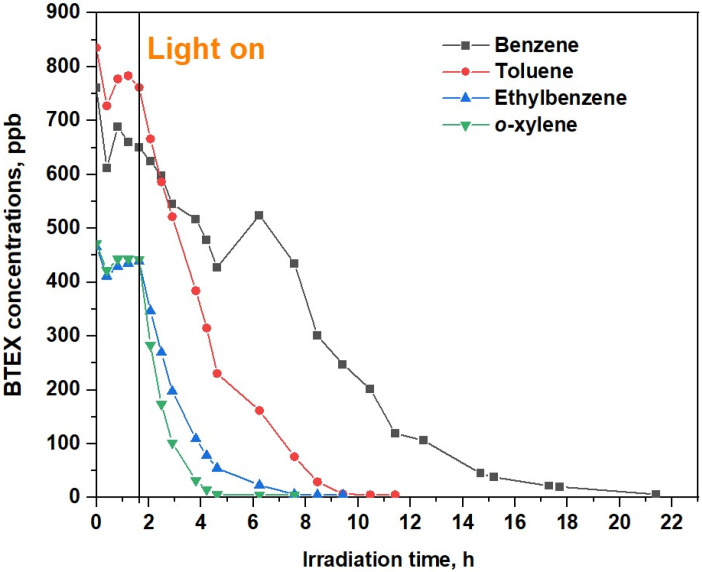
Evolution of the BTEX concentrations with time under recirculation over TiO_2_/volcanic glass.

Control tests conducted with a disabled photocatalytic module demonstrated the stability of toluene concentrations over a 5 h period (Figure S5). The degradation profiles of the BTEX over the TiO_2_/volcanic glass-based photocatalyst are present in Figure 1. The photocatalytic degradation of a mixture of gaseous BTEX was carried out at 20–22 °C and a relative humidity (RH) of 40%, under UV-A light with an intensity of 15 mW/cm^2^. Sequential BTEX degradation is shown in Figure 1 over a 24 h period.

**Figure 2:**
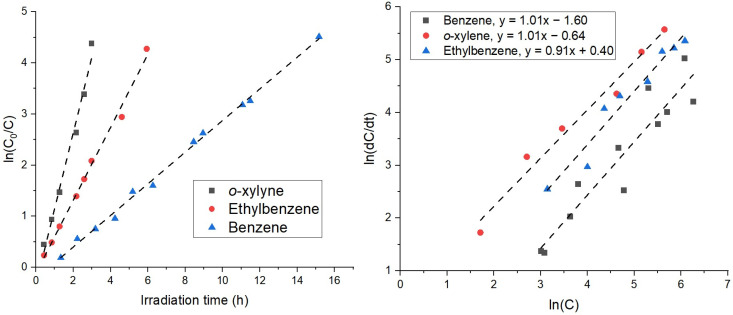
Pseudo-first-order kinetic plots of *o*-xylene, ethylbenzene, and benzene (for benzene after 7 h) undergoing photocatalytic oxidation.

Interestingly, the time series changes in toluene, ethylbenzene, and *o*-xylene (TEX) concentrations show that these compounds are oxidized at a faster rate (Figure 1), requiring 5–9 h for the complete removal of TEX from the gas phase, in contrast to benzene, which required approximately 21 h for complete degradation. The photodegradation kinetics of ethylbenzene and *o*-xylene were well-fitted to a pseudo-first-order reaction *ln*(C/C_0_) = −kt, where C_0_ represents the initial toluene concentration, C is the concentration at time t, and k is the degradation rate constant (h^−1^). By analyzing the fitting results presented in Figure 2, the rate constants, determined from the slopes of the straight lines, were 1.42 ± 0.08 h^−1^ and 0.71 ± 0.02, respectively, for ethylbenzene and *o*-xylene. In contrast, the oxidation of toluene and benzene does not follow a pseudo-first-order reaction. One potential explanation for this behavior is the production of benzene from the oxidation of toluene, ethylbenzene, and *o*-xylene (see, for example, [10,14]). Figure 1 clearly depicts the formation of benzene at the 7 h mark. 

The relative photocatalytic rates of organic compound decomposition were ascribed to the strength of the expected type of attractive force between the target compounds and the hydrated titania surface [20]. Authors using functional DFT calculations [21] have demonstrated that more than 50% of the adsorption energy for the gas-phase adsorption of BTEX on TiO_2_ arises from dispersion, with the remainder likely originating from the charge-induced polarization of the molecular electron density rather than from charge transfer. Based on polar moments, the selected BTEX compounds can be arranged in descending order of their molecular dipole moments: *o*-xylene > ethylbenzene > toluene > benzene. Considering the above, the sequential degradation of BTEX compounds can be attributed to the different types of attractive forces between BTEX and the catalyst surface—specifically weak dipole–dipole interactions for *o*-xylene, ethylbenzene, and toluene, while very weak dispersive forces dominate for benzene.

This sequential removal of BTEX compounds can be attributed to competitive adsorption, as previously noted by [9]. Additional evidence of this is that benzene begins to decompose in a pseudo-first-order reaction beyond 6 h, when about 99% of the *o*-xylene and ethylbenzene and 79% of the toluene are removed from the gas phase. This is shown in Figure 2, where the linear correlation of *ln*(dC/dt) as a function of *ln*(C) with a slope of *n* = 1.01 ± 0.12 is found for the benzene degradation profile data obtained after 6 h of photocatalytic oxidation in the BTEX mixture. The slope corresponds to the reaction order.

### 2.3. Hazard Quotients and Cancer Risk Estimates

The concentrations of volatile organic compounds in the atmosphere are subject to regulatory measures aimed at mitigating their impact on human health and the environment. Various agencies have established air quality guidelines, which provide recommended maximum values for specific pollutants in non-industrial environments and workplaces. Regulatory limits can vary depending on the geographical location and the agency responsible and are typically designed to address concerns related to air quality. In the United States, for instance, specific limits are defined as follows: the Threshold Limit Value (TLV) is issued by the American Conference of Governmental Industrial Hygienists (ACGIH), the Permissible Exposure Limits (PEL or OSHA PEL) are set by the Occupational Safety and Health Administration (OSHA), and the Recommended Exposure Limits (REL) are recommended by the United States National Institute for Occupational Safety and Health. It is important to note that the limits prescribed by different regulatory bodies may vary due to variations in the methodologies used to assess these limits. 

Another distinct aspect pertains to the regulation of indoor VOC concentrations. Presently, there are specific challenges associated with the implementation of nationally enforced standards for VOCs in non-industrial settings [13,22]. To evaluate indoor air quality, one can refer to USEPA-developed reference concentrations for inhalation exposure (RfC), Agency for Toxic Substances and Disease Registry (ATSDR)-developed minimal risk levels, or standards specific to other countries [23,24]. RfC standards provide estimates of daily human exposure, ensuring that air-borne exposure minimizes potential non-cancer health effects within a specified duration. In Kazakhstan, air quality is governed by hygienic standards applicable to atmospheric air in both urban and rural areas, as well as within industrial facilities [25]. These standards specify the upper limits and daily average maximum allowable concentrations of pollutants.

To assess the effectiveness of photocatalytic oxidation in minimizing the risk to human health, the Hazard Quotient (HQ) can be calculated over time, showing the potential for non-cancer health hazards resulting from exposure to a contaminant, taking into account the above-mentioned non-cancer health guidelines:

(1) $$HQ=\frac{C_{i}}{C_{i}^{ref}}$$

where HQ is the Hazard Quotient, $C_{i}$—Air Concentration (µg m^−3^ or ppb), and $C_{i}^{ref}$ = The Reference Concentration (µg m^−3^ or ppb). 

Once the HQ is calculated, it needs to be compared to a value of one. HQs less than one indicate a non-cancer hazard that should not be an issue. When a HQ is greater than one, the contaminants should be retained and an in-depth toxicological effects analysis conducted. In other words, a HQ above one means there is an exceedance of the non-cancer health guidelines. Additionally, it is worth noting that there are certain challenges associated with assessing hazard indexes for gas mixtures and evaluating the additive effects of exposure to multiple chemicals [11]. The United Technologies Research Center proposed a tolerance metric that defines a yardstick for measuring the tolerance of human beings to a given indoor air mixture [13].

In previous photocatalytic research, exposure limits derived for workplaces [10,11,12] are usually used as a reference concertation. This approach does not allow for an objective assessment of photocatalytic efficiency for intended indoor applications because exposure limits can be varied. Table 1 lists the BTEX exposure limits for residential areas and workplaces. Exposure limits for BTEX differ greatly; the most stringent regulatory requirements are given in ATSDR-developed minimal risk levels. At the same time, exposure limits for workplaces are much higher than the corresponding values of RfC for specified pollutants. Thus, using exposure limits developed for workplaces instead of limits for daily human exposure will lead to an underestimation of the hazard quotient. In our view, reevaluating the THI considering USEPA RfCs, instead of workplace-related limits, may result in higher THI values than those reported in prior research. Furthermore, as previously demonstrated by Mo et al. in 2009, the Health Risk Index (HRI) for the outlet consistently exceeded that of the inlet gas composition, implying the potential generation of more harmful by-products during the photocatalytic oxidation (PCO) process [12].

In our study, the RfC was used to evaluate changing air quality (HQ) during photocatalytic processes and was calculated as proposed in the study [11]:

(2) $$THI(t)=\sum_{i}^{\;}\frac{C_{i}(t)}{C_{i}^{ref}}\;\;$$

(3) $$MHQ(t)=\max\;\left\{\frac{C_{i}(t)}{C_{i}^{ref}};\sum_{i}^{\;}\frac{C_{i}(t)}{C_{i}^{ref}}\right\}\;\;\;\;\;$$

Table 1 illustrates that among the BTEX compounds, the most stringent regulatory levels are associated with benzene, with its RfC being several times lower than that of the other members. Notably, Kazakhstan’s regulatory documents specify a relatively high exposure limit for benzene, with the maximum permissible concentration in the atmospheric air of populated areas set at 100 μg/m^3^ for 24 h, which is about three times higher than the USEPA RfC. In comparison, atmospheric benzene concentrations in Almaty, Kazakhstan have been reported up to 341 μg/m^3^ [26]. Furthermore, our previous review [8] has demonstrated that the primary hazardous gas-phase intermediates formed during toluene PCO are benzene and formaldehyde, both of which are classified by the International Agency for Research on Cancer (IARC) as Group 1 carcinogenic compounds. Consequently, we conducted an assessment to estimate cancer risk using the Inhalation Unit Risk (IUR) values presented in Table 1.

**Table 1:** Comparison of inhalation exposure limits for formaldehyde, benzene, and toluene.

Pollutant	Benzene CAS 71-43-2	Toluene CAS 108-88-3	Ethylbenzene CAS 100-41-4	Xylenes CAS 1330-20-7 CAS 106-42-3 CAS 108-38-3 CAS 95-47-6	Ref.
DIRECTIVE 2008/50/EC	5 μg/m^3^ Calendar year	-	-	-	[27]
EPA-Derived Reference Concentration for Inhalation Exposure (RfC)	3 × 10^−2^ mg/m^3^	5 mg/m^3^	1 mg/m^3^	XYLENES 1 × 10^−1^ mg/m^3^	[28]
ACGIH, TLV	TWA 0.5 ppm STEL 2.5 ppm	TWA 20 ppm	TWA 20 ppm		[29]
NIOSH, REL	Ca TWA 0.1 ppm ST 1 ppm	TWA 100 ppm (375 mg/m^3^) ST 150 ppm (560 mg/m^3^)	TWA 100 ppm (435 mg/m^3^) ST 125 ppm (545 mg/m^3^)	For each xylenes separately TWA 100 ppm (435 mg/m^3^) ST 150 ppm (655 mg/m^3^)	[30]
OSHA, PEL	TWA 1 ppm ST 5 ppm	TWA 200 ppm C 300 ppm 500 ppm (10 min maximum peak)	TWA 100 ppm (435 mg/m^3^)	For each xylenes separately TWA 100 ppm (435 mg/m^3^)	[31]
ATSDR-Developed Minimal Risk Levels	0.009 ppm (acute) 0.006 ppm (int) 0.003 ppm (Chr.)	2 ppm (acute) 1 ppm (Chr.)	5 ppm (acute) 2 ppm (int) 0.06 ppm (Chr.)	XYLENES, MIXED 2 ppm (acute) 0.6 ppm (int) 0.05 ppm (Chr.)	[23]
Maximum permissible concentration—KZ	0.1 mg/m^3^ (24 h) 0.3 mg/m^3^ single max	0.6 mg/m^3^ single max	0.02 mg/m^3^ single max	XYLENES, MIXED 0.2 mg/m^3^	[25]
Inhalation Unit Risk	7.8 × 10^−6^ per µg/m^3^				[28]

**Figure 3:**
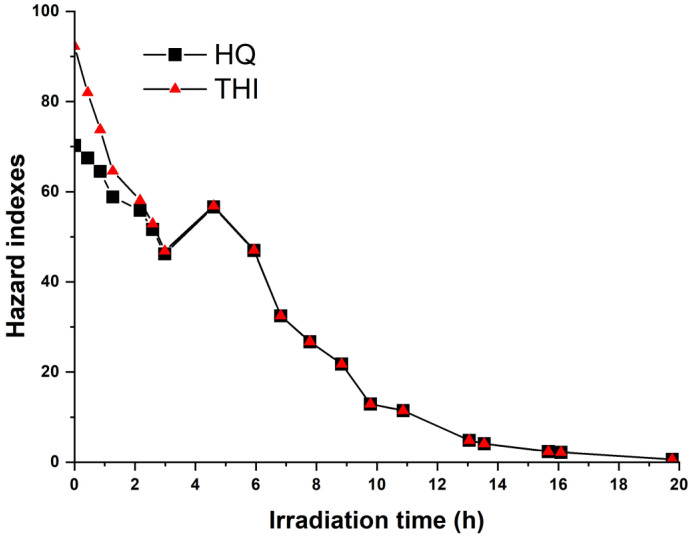
The hazard indexes during the BTEX PCO over a TiO_2_/volcanic glass catalyst.

Figure 3 illustrates the time-dependent change in the calculated Total Hazard Index (THI) and Hazard Quotient (HQ) during the photocatalytic degradation of BTEX over TiO_2_/volcanic glass. It is evident that both HQ and THI values consistently remained higher than the permissible level of 1.0 during the PCO process; only at the end of the photocatalytic treatment, beyond 16 h, the THI reach a level below the permissible level of 1.0. The calculated HQ showed that the most substantial contribution to the THI was due to benzene, while the contribution of toluene and ethylbenzene to the THI was negligible. It is evident from Figure 3 that the THI value increased from 47 to 57 after 3 h of photocatalytic oxidation, which is attributed to the generation of benzene in the system. Thus, the mixture effect that delays the removal of benzene from the gas phase can result in the reduced removal efficiency during air purification by PCO technology. 

At present, a multitude of methodologies for calculating carcinogenic risk have been developed [32]. In this study, the assessment of cancer risk associated with specific compounds was conducted using the Inhalation Unit Risk (IUR) values, as per the following equation [24]:

(4) $$CR=IUR\times EC=IUR\times \frac{CA\times ET\times EF\times ED}{AT}$$

where CR is the Cancer Risk; EC (µg/m^3^) is the exposure concentration; CA (µg/m^3^) is the contaminant concentration in the air; ET (hours/day) is the exposure time; EF (days/year) is the exposure frequency; ED (years) = exposure duration; and AT (lifetime in years × 365 days/year × 24 h/day) is the averaging time. 

With a conservative approach, wherein the exposure concentration (EC) is set to be equal to the contaminant concentration (CA), the calculated cancer risk (CR) associated with the presence of benzene can result in a CR value range from 1.6 × 10^−2^ to 4.1 × 10^−4^, surpassing the threshold of 10^−6^. Consequently, in scenarios involving such concentrations, it is crucial to conduct a comprehensive toxicological effects analysis. This analysis should take into account various factors, including exposure time, frequency, duration for each receptor being assessed, the averaging period for exposure, and other relevant considerations.

Obviously, BTEX by-products will also affect the quality of treated air. In this study, the formation of phenol and benzaldehyde in the gas phase remained below the detection limit of ~4 ppb. A review by Tulebekov et al. [8] highlighted benzene and formaldehyde as major hazardous gas-phase intermediates formed during the PCO of aromatic compounds. However, it was not feasible to detect formaldehyde using solid-phase microextraction in the present study. Future evaluation of air quality should encompass the study of formaldehyde formation for a more comprehensive understanding of its potential impact on human health. Furthermore, it is essential to identify material activity descriptors to establish relationships between catalyst physicochemical properties and performance. This approach can significantly save time and costs, facilitating the design and development of efficient catalysts [33].

## 3. Materials and Methods

### 3.1. Experimental Set-Up

**Figure 4:**
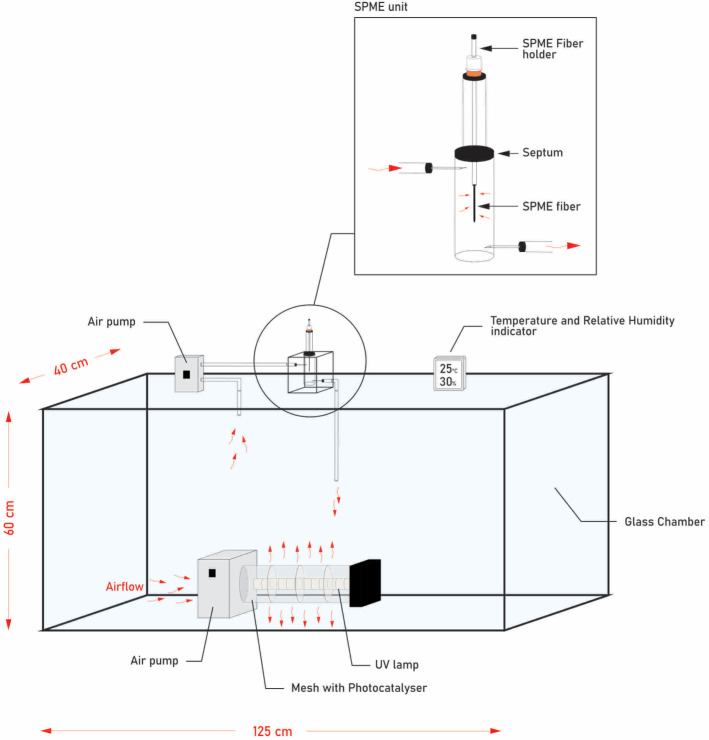
Schematic Illustration of the Experimental Setup for BTEX Photodegradation.

Figure 4 depicts a schematic illustration of the photocatalytic oxidation setup employed in this study. The primary components of the apparatus included: (1) The photocatalytic reactor module; (2) The sampling module for solid-phase microextraction (SPME); (3) The test chamber.

The photocatalytic oxidation (PCO) of BTEX was conducted in a continuous-flow mode using an annular reactor with the following specifications: an inner diameter of 8 cm, a length of 15 cm, and an inner surface area of 900 cm^2^. In this study, the loading of photocatalyst onto the catalyst support followed the procedure outlined by [13], with some modifications. TiO_2_-suspended powder was spread uniformly over the volcanic glass mesh to achieve a loading of ca. 0.5 mg/cm^2^. The solution was prepared by suspending titania (5% by weight) in distilled water. The specific surface area of the reactor element changed from ~1 m^2^/g to 78.9 m^2^/g after loading the catalyst onto the support surface. The TiO_2_ mixture contained anatase and rutile nanopowder with a particle size of less than 100 nm and a stated purity of 99.5% TiO_2_ (Sigma-Aldrich, St. Louis, MO, USA), the BET surface area of which was determined to be 78.9 m^2^/g. Refinement using the Le Bail method was conducted to determine the phase composition and unit cell parameters. This analysis was carried out utilizing the GSAS-II software package (https://subversion.xray.aps.anl.gov/trac/pyGSAS/wiki/ProxyInfo accessed on 3 November 2023) [34]. 

The selection of photocatalytic oxidation parameters, including humidity, UV type and intensity, and flow rate, was carefully conducted to align with the conditions anticipated for use in practical photocatalytic air purifiers [13]. UV illumination was generated by a 36 W fluorescent tube (Philips, Pila, Poland), which resulted in a uniform-intensity of 15 mW/cm^2^, with a spectral peak centered on 371 nm (spectrum in Supplementary Materials Figure S6) at the catalyst surface. A UV-A fluorescent lamp was axially located inside the annular reactor. The test was performed at a 55 m^3^/h total flow rate, with an approximatively similar residence time of 0.01 s within the illuminated zone of the reactor.

A test chamber with parallelepipedic dimensions of 40 cm length, 125 cm width, and 60 cm height, constructed from borosilicate glass, was employed for the measurement of toluene and its degradation products (refer to Figure 4). The test chamber was equipped with temperature and relative humidity control sensors. This chamber was directly linked to a solid-phase microextraction sampling unit. The inside of the chamber was filled with high purity gas mixtures of 20% O_2_/N_2_. After a measured volume of benzene, toluene, ethylbenzene, and *o*-xylene was introduced into the test chamber through syringe injections, the chamber was then homogenized to achieve an adsorption equilibrium. Subsequently, the photocatalytic module was activated. 

### 3.2. BTEX Concentration Measurements

Gas-phase concentration measurements of BTEX in the air chamber were carried out using solid-phase microextraction (SPME), known for its low limit of detection for many compounds. Low detection limits are possible for quantifying volatile organic compounds (VOCs), with detection limits for benzene, toluene, ethylbenzene, and *o*-xylene at 0.5 µg m^−3^, 1.6 µg m^−3^, 0.2 µg m^−3^, and 0.1 µg m^−3^, respectively [35]. Briefly, the procedure involved the equilibration of an 80 μm divinylbenzene/carbon wide-range/polydimethylsiloxane (DVB/CWR/PDMS) fiber (Product No. 5191-5874) in an air-swept chamber. The extraction was conducted at 22–25 °C (room temperature) for a duration of 5 min, followed by immediate analysis using gas chromatography–mass spectrometry (GC–MS). The fiber was preconditioned as per the manufacturer’s instructions by injection into the GC injector port. Calibration curves (R^2^ = 0.99) were established for all compounds prior to each experiment.

Instrumental analysis used a Thermo Trace GC coupled to a Polaris Q Ion trap Mass-spectrometer (Thermo Finnigan, Milan, Italy). Chromatographic separation employed a 30 m × 0.25 mm × 0.25 µm film-thickness capillary column (DB-HeavyWAX, Agilent Technologies, Santa Clara, CA, USA, p/n: 122-7132). Oven temperatures began at 60 °C and were held for 1.5 min, followed by an increase of 200 °C per minute until it reached 150 °C, followed by a further ramping at a rate of 100 °C per minute up to 250 °C, where it was held for 1 min. Compound identification was conducted by comparing the mass spectra and retention times of the analyte samples to those of corresponding standards. Calibration standards were prepared by evaporating pure solvent in containers of a known volume, equilibrating and diluting the equilibrated vapor over a linear calibration range of between 5 and 1000 ppb benzene, toluene, ethylbenzene, and *o*-xylene. To evaluate the accuracy of the measurements, following every fifth measurement, analysis of the in-house reference standard was conducted. This standard represents a mixture of BTEX compounds, benzaldehyde, and phenol, each with a concentration of 50 ppb. The concentrations of the determined compounds deviated by less than 15% from the reference values. Method precision was evaluated through the analysis of duplicate samples, resulting in a relative standard deviation (%RSD) of less than 10%. The method detection limits, determined through replicate analysis of blanks, were found to be ~2 ppb for benzene, toluene, ethylbenzene, and *o*-xylene, and ~4 ppb for benzaldehyde and phenol.

## 4. Conclusions

This study, employing solid-phase microextraction in conjunction with GC–MS, demonstrates that the sequential removal of BTEX occurred during the photocatalytic oxidation of the BTEX over a TiO_2_/volcanic glass catalyst in a test chamber. The mixture effect, which delays the removal of benzene from the gas phase, can indeed lead to a decrease in the quality of the purified air. The conservative approach to calculating the Total Hazard Index (THI) in the process of photocatalytic oxidation indicates that it remains higher than acceptable values, mainly due to the presence of benzene. Furthermore, when considering the cancer risk associated with inhalation exposure, it may exceed the threshold of 10^−6^. Hence, it is imperative to incorporate monitoring of benzene formation and perform an in-depth toxicological effects analysis when carrying out photocatalytic purification of BTEX in indoor applications. This is particularly essential given benzene’s classification as a carcinogenic compound and the strict regulatory levels that have been upheld over recent decades.

## Data Availability

Some data are presented in the Supplementary Materials. Other data are available from the authors if needed for review.

## Supplementary Materials

The following supporting information can be downloaded at: https://www.mdpi.com/article/10.3390/molecules28248119/s1, Figure S1: Observed (blue line) and calculated (green line) X-ray diffraction patterns and their difference profile (turquoise line: Io-Ic) for the X-ray diffraction pattern of TiO_2_ using the Le Bail method by GSAS-II program. Figure S2: Observed (blue line) and calculated (green line) X-ray diffraction patterns and their difference profile (turquoise line: Io-Ic) for the X-ray diffraction pattern of volcanic glass using the Le Bail method by GSAS-II program. Figure S3: TG/DSC curves for volcanic glass sample mesuread in dry nitrogen using a thermoanalyzer (NETZSCH 449F3A−0372−M) over the temperature range of 20–1000 °C. Figure S4: An IR spectrum was conducted for a sample of volcanic glass substrate, encompassing wavenumber values ranging from 450 to 4000 cm^−1^. Figure S5: Evolution of the toluene concentration with time under recirculation over TiO_2_ /volcanic glass. Figure S6: Emission spectrum of the 36 W fluorescent tube (Philips-PL-L-36W/10/4).
