# Supplementary material for: Human Health Risk Assessment of the Photocatalytic Oxidation of BTEX over TiO2/Volcanic Glass
Source: Molecules. 2023 Dec 15;28(24):8119. doi: 10.3390/molecules28248119 (PMC10745685; doi:10.3390/molecules28248119)
Supplement: Supplementary file 1 [file molecules-28-08119-s001.zip › molecules-2726502-supplementary.pdf]

Supporting Information

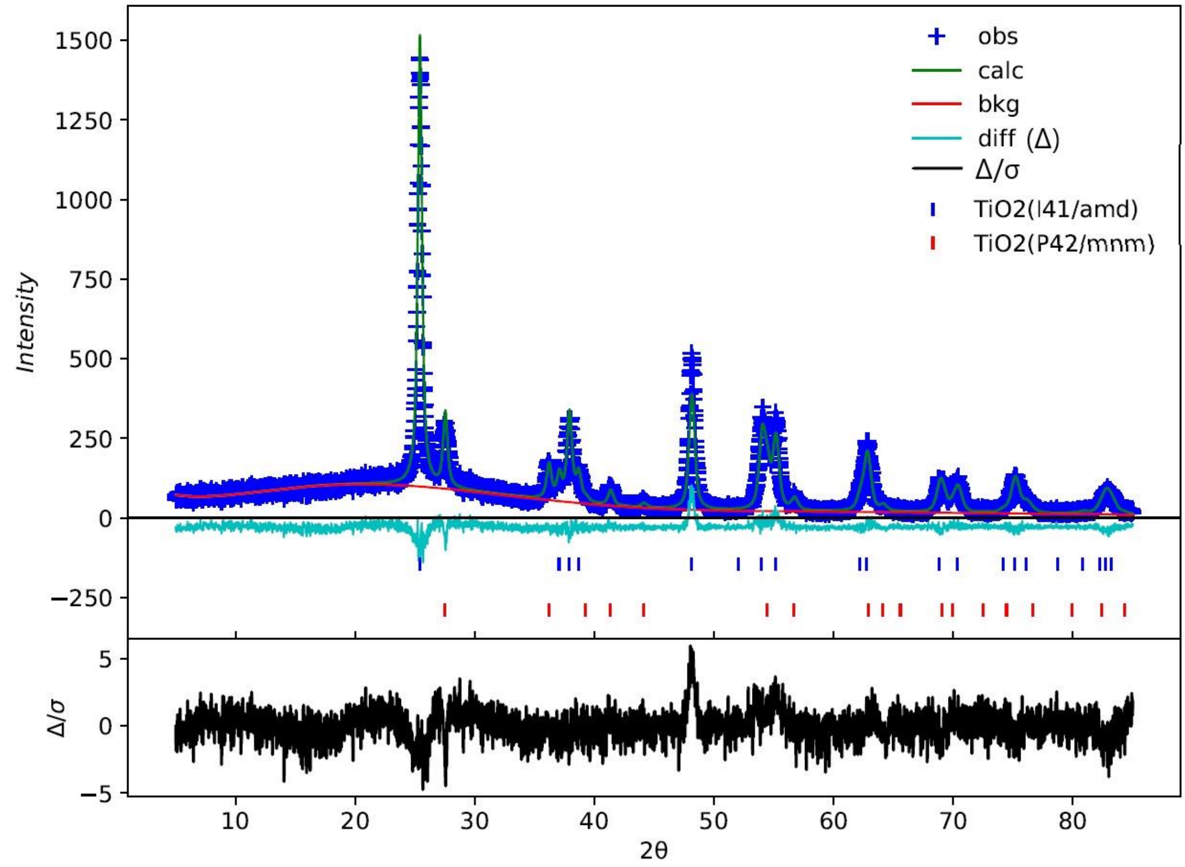

Figure S1. Observed (blue line) and calculated (green line) X-ray diffraction patterns and their difference profile (turquoise line: Io-Ic) for the X-ray diffraction pattern of TiO_2 using the Le Bail method by GSAS-II program. The lowest curve (black line) shows the difference between the observed and calculated powder diffraction patterns divided by standard deviation σ(Io) of observed pattern plotted against 2θ.

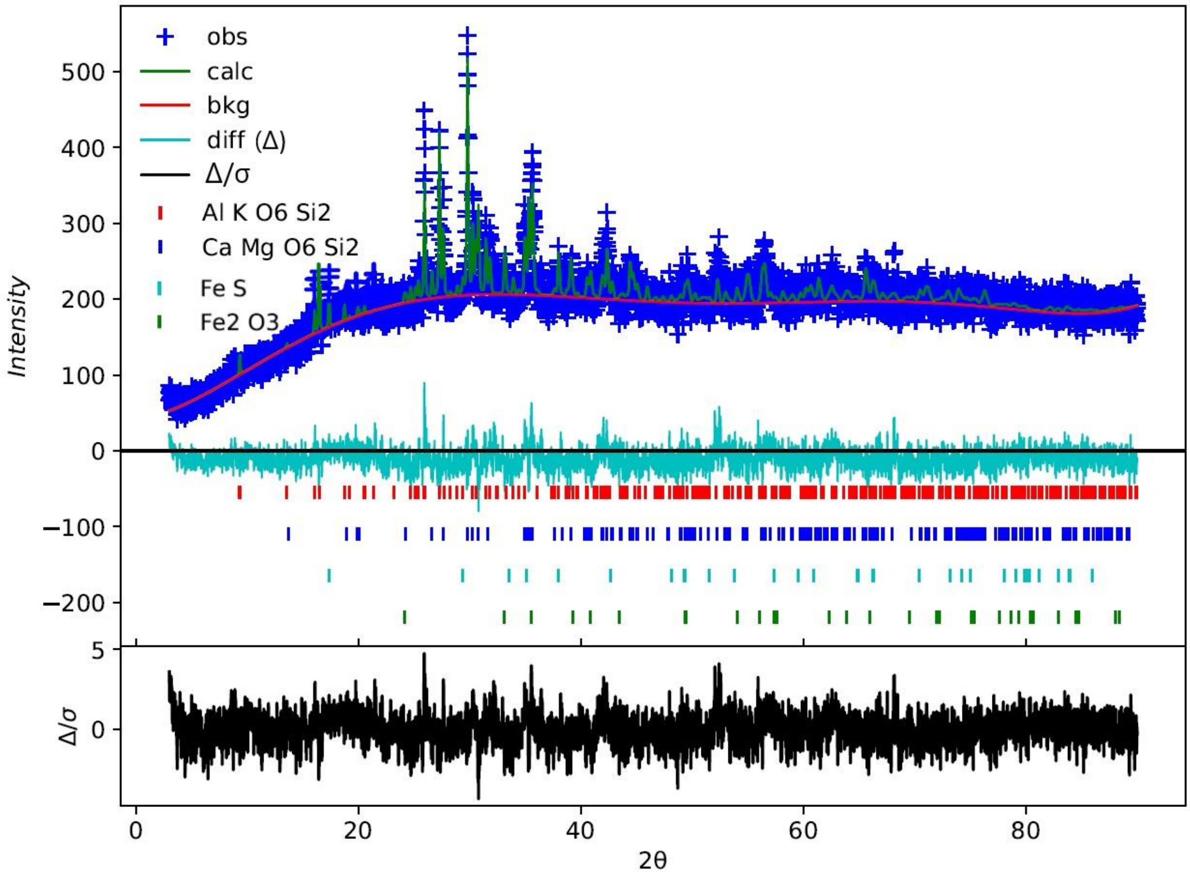

Figure S2. Observed (blue line) and calculated (green line) X-ray diffraction patterns and their difference profile (turquoise line: Io-Ic) for the X-ray diffraction pattern of volcanic glass using the Le Bail method by GSAS-II program. The lowest curve (black line) shows the difference between the observed and calculated powder diffraction patterns divided by standard deviation σ(Io) of observed pattern plotted against 2θ.

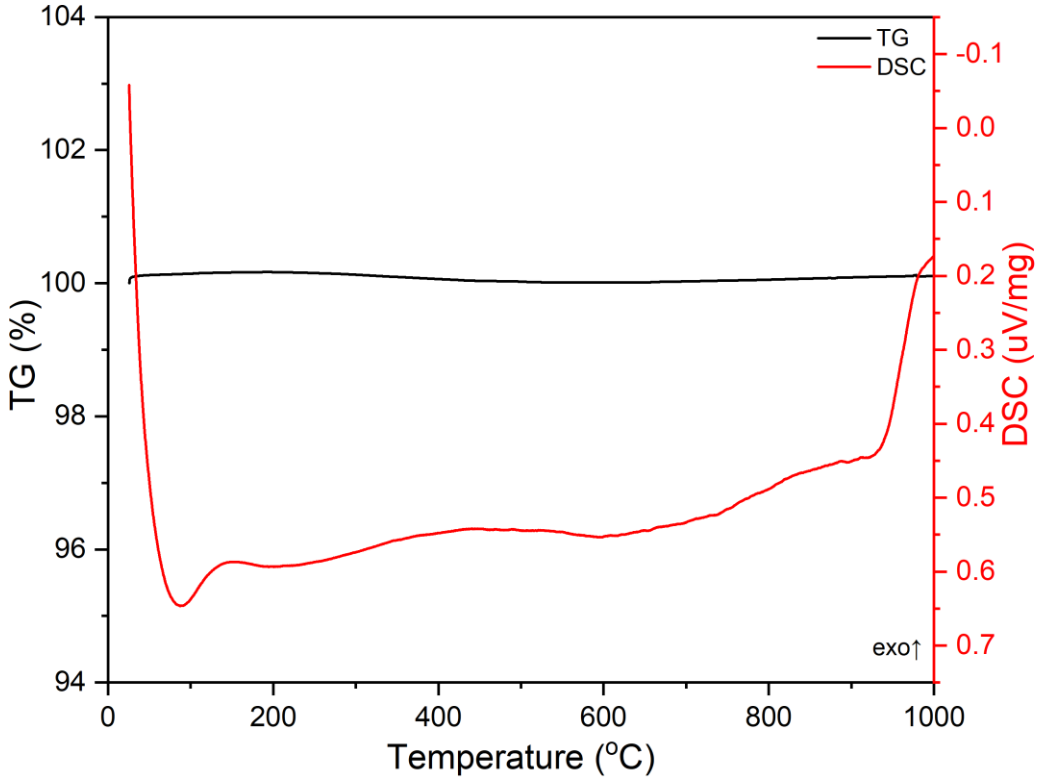

Figure S3. TG/DSC curves for volcanic glass sample mesuread in dry nitrogen using a thermoanalyzer (NETZSCH 449F3A−0372−M) over the temperature range of 20- 1000 ◦C. The parameters during measurements were as follows: crucible of DSC/TG pan Al2O3; heating rate 10 K min^{-1}

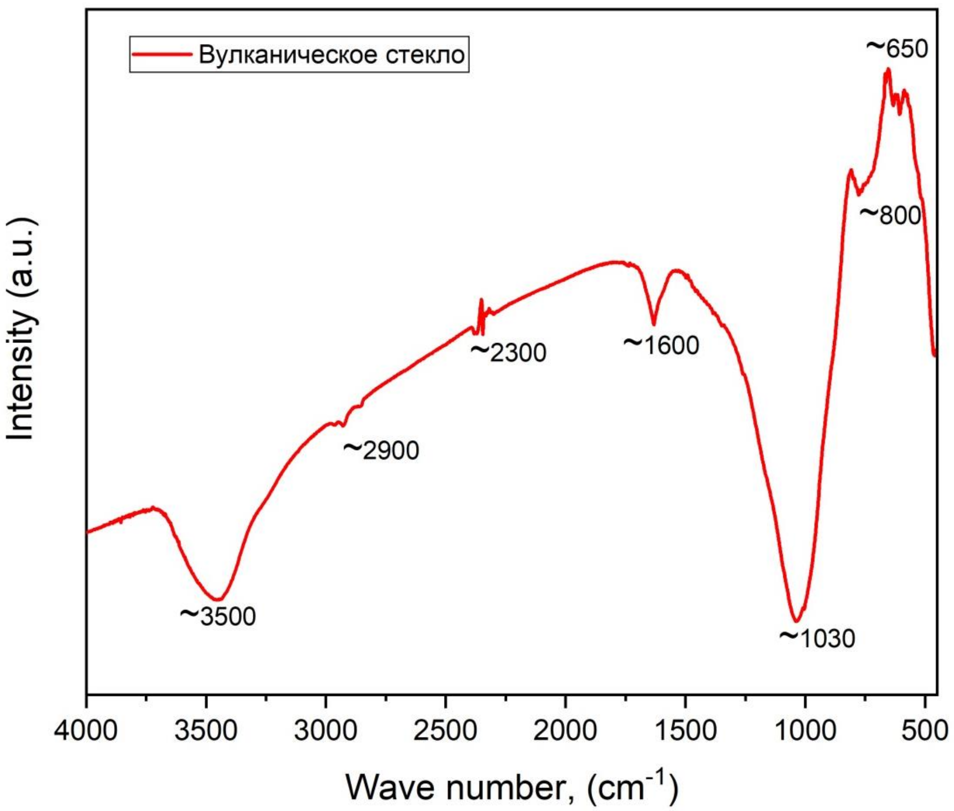

Figure S4. An IR spectrum was conducted for a sample of volcanic glass substrate, encompassing wavenumber values ranging from 450 to 4000 cm^{-1}. Through factor group analysis of the vibrations associated with Si–O–Si and Al–O–Si bonds within the glass sample, the IR spectrum reveals several significant features. These include a principal absorption peak at 1035 cm^{-1}, a relatively strong peak at approximately 1600 cm^{-1}, and a weaker peak around 800 cm^{-1}. Additionally, there are minor peaks observed at approximately 650 cm^{-1}, 2300 cm^{-1}, and 2900 cm^{-1}.

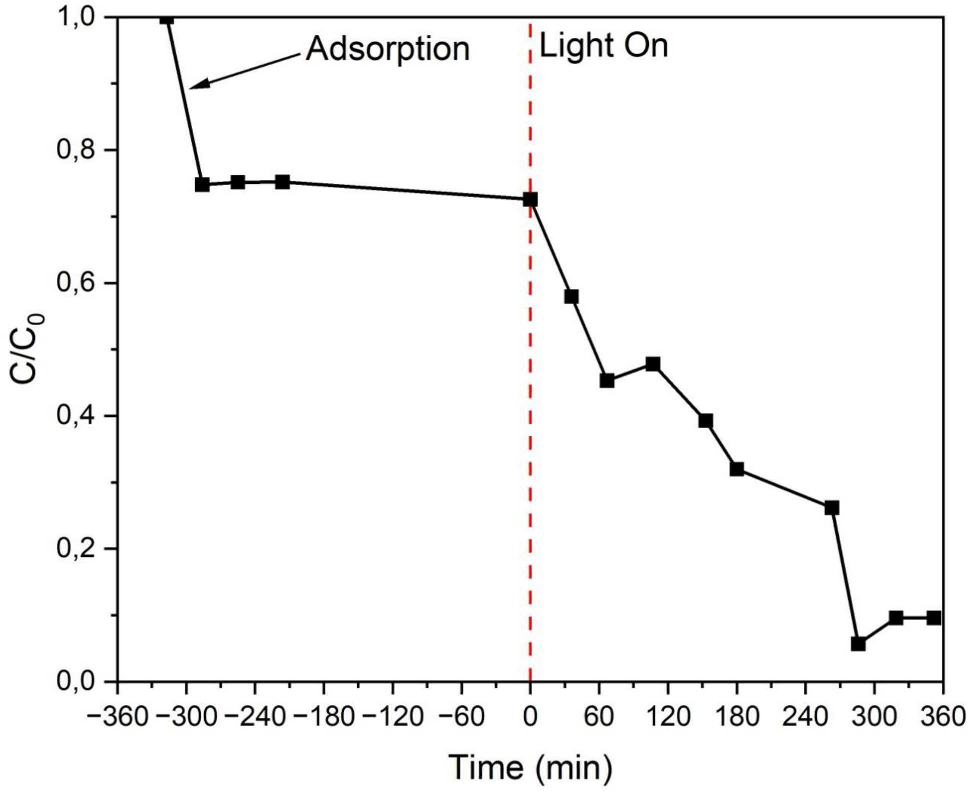

Figure S5. Evolution of the toluene concentration with time under recirculation over TiO_2 /volcanic glass

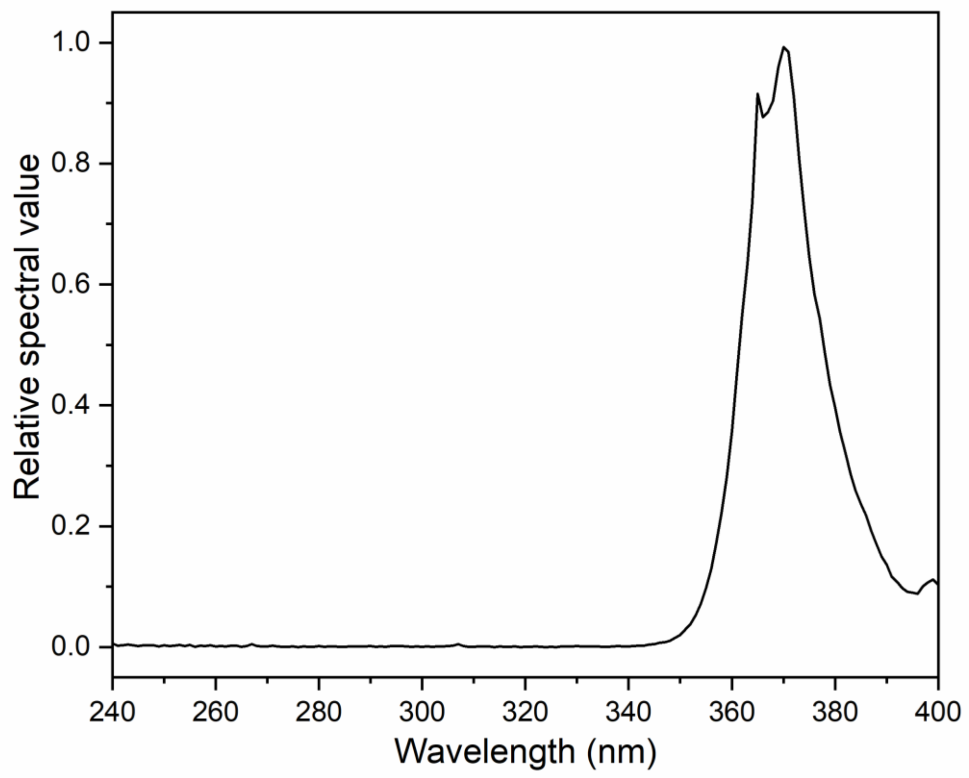

Figure S6. **Emission spectrum of the 36W fluorescent tube (Philips-PL-L-36W/10/4)** with a spectral peak centered on 371 nm. The lamp emits with an irradiance:

Items	value	Items	value	Items	value
Euvb(mW/cm2)	3.3117E-2	Euvc(mW/cm2)	6.3672E-2	Euva(mW/cm2)	1.5051E1
Lp(nm)	370.8	HW(nm)	16.2	Ee(240.0~300.0)(mW/cm2)	8.0666E-2
